# Machine learning approaches to the human metabolome in sepsis identify metabolic links with survival

**DOI:** 10.1186/s40635-022-00445-8

**Published:** 2022-06-17

**Authors:** Leah B. Kosyakovsky, Emily Somerset, Angela J. Rogers, Michael Sklar, Jared R. Mayers, Augustin Toma, Yishay Szekely, Sabri Soussi, Bo Wang, Chun-Po S. Fan, Rebecca M. Baron, Patrick R. Lawler

**Affiliations:** 1 Peter Munk Cardiac Centre, University Health Network, Toronto, Canada; 2 Department of Medicine, University of Toronto, Toronto, Canada; 3 Beth Israel Deaconess Medical Center, Harvard Medical School, Boston, MA USA; 4 Rogers Computational Program, Ted Rogers Centre for Heart Research, University of Toronto, Toronto, Canada; 5 Department of Medicine, Stanford University School of Medicine, Stanford, CA USA; 6 Interdepartmental Division of Critical Care Medicine, University of Toronto, Toronto, Canada; 7 Department of Anesthesia, St. Michael’s Hospital, Toronto, Canada; 8 Department of Chemistry and Chemical Biology, Harvard University, Cambridge, MA USA; 9 Division of Pulmonary and Critical Care Medicine, Brigham and Women’s Hospital, Harvard Medical School, Boston, MA USA; 10 Division of Cardiology, Tel Aviv Sourasky Medical Center, Tel Aviv, Israel; 11 Vector Institute for Artificial Intelligence, Toronto, ON Canada; 12 Department of Medical Biophysics, University of Toronto, Toronto, ON Canada; 13 Peter Munk Cardiac Center, Toronto General Hospital, RFE3-410, 190 Elizabeth St., Toronto, ON M5G 2C4 Canada

**Keywords:** Artificial intelligence, Machine learning, Metabolism, Metabolomics, Sepsis

## Abstract

**Background:**

Metabolic predictors and potential mediators of survival in sepsis have been incompletely characterized. We examined whether machine learning (ML) tools applied to the human plasma metabolome could consistently identify and prioritize metabolites implicated in sepsis survivorship, and whether these methods improved upon conventional statistical approaches.

**Methods:**

Plasma gas chromatography–liquid chromatography mass spectrometry quantified 411 metabolites measured ≤ 72 h of ICU admission in 60 patients with sepsis at a single center (Brigham and Women’s Hospital, Boston, USA). Seven ML approaches were trained to differentiate survivors from non-survivors. Model performance predicting 28 day mortality was assessed through internal cross-validation, and innate top-feature (metabolite) selection and rankings were compared across the 7 ML approaches and with conventional statistical methods (logistic regression). Metabolites were consensus ranked by a summary, ensemble ML ranking procedure weighing their contribution to mortality risk prediction across multiple ML models.

**Results:**

Median (IQR) patient age was 58 (47, 62) years, 45% were women, and median (IQR) SOFA score was 9 (6, 12). Mortality at 28 days was 42%. The models’ specificity ranged from 0.619 to 0.821. Partial least squares regression-discriminant analysis and nearest shrunken centroids prioritized the greatest number of metabolites identified by at least one other method. Penalized logistic regression demonstrated top-feature results that were consistent with many ML methods. Across the plasma metabolome, the 13 metabolites with the strongest linkage to mortality defined through an ensemble ML importance score included lactate, bilirubin, kynurenine, glycochenodeoxycholate, phenylalanine, and others. Four of these top 13 metabolites (3-hydroxyisobutyrate, indoleacetate, fucose, and glycolithocholate sulfate) have not been previously associated with sepsis survival. Many of the prioritized metabolites are constituents of the tryptophan, pyruvate, phenylalanine, pentose phosphate, and bile acid pathways.

**Conclusions:**

We identified metabolites linked with sepsis survival, some confirming prior observations, and others representing new associations. The application of ensemble ML feature-ranking tools to metabolomic data may represent a promising statistical platform to support biologic target discovery.

**Supplementary Information:**

The online version contains supplementary material available at 10.1186/s40635-022-00445-8.

## Background

Mortality from sepsis remains unacceptably high [1], compelling an ongoing search for novel disease mediators which may represent therapeutic targets. Metabolic alterations have represented an under-explored pathophysiologic axis in sepsis [2, 3]. Unbiased, high-dimensional molecular platforms for profiling hundreds of circulating metabolites in concert (metabolomics) [4, 5] have opened opportunities for data-driven, systems biology approaches to clinical risk prediction as well as the search for potential novel therapeutic targets in humans [6].

Although the application of metabolomics in critical care research has increased over recent years [2, 7–9], optimal statistical analytic approaches to such high-dimensional data remain uncertain, particularly when there is a large imbalance between the number of metabolites profiled relative to the number of clinical events. Conventional statistical methods such as logistic regression may have important limitations when applied to data with high degrees of internal correlation (including the metabolome), missingness, subclass heterogeneity, and imbalance between exposures (metabolites) and outcomes—frequent challenges in high-dimensional human biologic data in critically cohorts. Analytic approaches using machine learning (ML), a subset of artificial intelligence, may overcome some of these challenges [10]. Such approaches have recently been successfully applied to metabolomics data with a focus on building clinical prediction models [11]. However, less focus has been placed on how robustly and consistently ML could enhance biologic discovery through statistical mining of the metabolome to identify metabolites may show the strongest links with clinical outcomes.

In the present study, we hypothesized that parallel and ensemble ML methods could facilitate identification of individual metabolites potentially implicated in sepsis survivorship using gas chromatography–liquid chromatography (GC/LC) mass spectrometry profiling of the human metabolome. Our two complementary aims were: (1) biologically, to uncover metabolite signals associated with mortality in sepsis; and (2) methodologically, to determine to what extent metabolite selection and prioritization through ML provided consistent, robust identification of metabolites despite a small cohort size, and whether these methods enhanced metabolite–outcomes links beyond conventional logistic regression. This study extends prior analyses from this cohort [12, 13] which employed conventional statistical methods including logistic regression, now comparing observations from ML approaches with those from the conventional statistical approaches.

## Methods

### Study cohort

The study population consisted of 60 adult patients with sepsis—30 of whom also had acute respiratory distress syndrome (ARDS)—admitted to the Medical Intensive Care Unit (ICU) at Brigham and Women’s Hospital in Boston, MA, USA (Registry of Critical Illness; RoCI), as previously described. [12] The study was approved by a local institutional ethics board. All subjects provided informed consent. Patients were enrolled in the larger RoCI cohort study between September, 2008 and May, 2010. In a subset of 225 patients, interleukin (IL)-18 levels were analyzed in a separate study of inflammasome-regulated cytokines in acute lung injury​ [12]. As a follow-up to this study, RoCI patients were selected for metabolomic profiling on the basis of whether ARDS complicated sepsis or not, and whether IL-18 was elevated or not (sepsis patients with low IL-18 levels and ARDS with high IL-18 levels, were included). This initial metabolomics study identified pathway analytes of interest that were independently validated in a separate critical illness cohort (the CAPSOD cohort). [14]. No outcomes enrichment nor selection on the basis of metabolite features was undertaken. Herein, we set out to evaluate differential analytic approaches to this dataset.

### Plasma metabolomics

Plasma was obtained from patients within 72 h of ICU admission. Gas and liquid chromatography and mass spectrometry was performed by Metabolon, Inc., as previously described [12]. Briefly, blood samples were collected in EDTA-coated blood collection tubes within 72 h of ICU admission and processed within 4 h after collection. The liquid chromatography/mass spectrometry (LC/MS) portion of the platform was performed on a Waters ACQUITY UPLC and a Thermo-Finnigan LTQ mass spectrometer, consisting of an electrospray ionization (ESI) source and linear ion-trap (LIT) mass analyzer. The gas chromatography/mass spectrometry (GC/MS) portion of the platform was performed on a Thermo-Finnigan Trace DSQ fast-scanning single-quadrupole mass spectrometer using electron impact ionization. Extracts were reconstituted with water and methanol. Data from 411 metabolites were available. Drug metabolites were excluded from this analysis.

### Statistical methods

Metabolites were pre-processed, filtered, and imputed where necessary (Additional file 1: Methods). This included a log10 transformation. Metabolites with > 10% of values below the lower detection limit were excluded from the analysis. We fit 7 ML and logistic regression models to predict mortality and then extracted the individual metabolites which contributed to the most accurate prediction models, viewing this as a measure of association with death. We performed multiple diverse ML methods (random forests, support vector machines, random *k*-nearest neighbors, nearest shrunken centroids, adaptive bagging/boosting, and Lasso regression), conventional bilinear factor models (partial least squares-discriminant analysis [PLS-DA]), as well as penalized logistic regression, predicting mortality using the post-processed metabolomics data. Models were trained under the precision recall (PR) curve using 50 repeats of fivefold cross-validation. Training under the PR curve was undertaken given theoretical advantages in imbalanced datasets, including providing a better assessment of the performance of a classifier on mortality [15]. Model sensitivity was assessed. For comparison, under the PR curve, a no skill classifier (one that performs no better than classifying all patients as positive) has an area under the PR curve equal to the observed event rate in the study population (0.42).

In the primary analysis, innate feature selection and ranking tools of ML models were used to prioritize metabolites (Additional file 1: Methods). The outcome used for prioritization was prediction of 28-day mortality. The top metabolites contributing to successful predictive model generation were compared across each method. An ensemble ranking procedure hybridizing multiple ML methods run in series provided a summary ranking of metabolites [16], and the variables with ensemble importance scores ≥ 0.5 were identified. This method performs iterative and parallel ML steps, and ranks top features (metabolites) based on the strength and consistency with which they are linked with the outcome. For each fitted model, we reported the metabolites that were selected/ranked using each model’s implicit selection/ranking procedure. We assessed the consistency between these different sets of selected (ranked) metabolites. We also assessed the performance of each of these ML models. In view of obtaining a more robust final set of important metabolites, we aimed to utilize the information obtained by each of these models. Thus, we created a score that represented the percentage of times that a metabolite was selected/ranked highly across all ML models.

All analysis was conducted in R *v*3.3.2 using packages caret (6.0–79), earth (4.6.2), spls (2.2–2), klaR, randomForest (4.6–14), RWeka (0.4–38), fastAdaboost (1.0.0), adabag (4.2), plyr (1.8.4), sparseLDA (0.1–9), glmnet (2.0–16), Matrix (1.2–14), gbm (2.1.3), pamr (1.55), and kernlab (0.9–26).

## Results

### Patient cohort

Median (IQR) patient age was 58 (47, 62) years and 45% of patients were women (Table 1). Median (IQR) SOFA and APACHE II scores were 9 (6, 12) and 30 (23, 37), respectively. Mortality at 28 days was 42%. Patients who died had higher illness severity scores (SOFA, *p* = 0.004; APACHE II, *p* = 0.02), more frequently presented with acute respiratory failure (*p* = 0.03) and ARDS (*p* < 0.001, as well as had a prior history of malignancy (*p* < 0.001).

**Table 1:** Baseline patient demographics

	Overall (*n* = 60)	Survived (*n* = 35)	Died (*n* = 25)	*p*-value*
Age, years	58 (47,62)	53 (46, 63)	62 (48, 67)	0.33
Women	27 (45%)	17 (49%)	10 (40%)	0.51
Race/ethnicity				0.41
White	53 (88%)	29 (83%)	22 (88%)	
Black	3 (5%)	4 (11%)	1 (4%)	
Hispanic	3 (5%)	2 (6%)	1 (4%)	
Asian	1 (2%)	0 (0%)	1 (4%)	
Comorbidities				
Diabetes	12 (20%)	8 (23%)	4 (16%)	0.44
Malignancy	23 (48%)	10 (29%)	19 (76%)	< 0.001
CKD**	16 (27%)	6 (18%)	10 (40%)	0.06
Liver disease	5 (8%)	3 (9%)	2 (8%)	0.91
COPD	7 (12%)	4 (11%)	3 (12%)	0.95
Illness severity				
ARDS	30 (50%)	12 (34%)	18 (72%)	< 0.001
Respiratory failure	51 (85%)	27 (77%)	24 (96%)	0.03
SOFA score	9 (6, 12)	8 (5, 10)	11 (7, 15)	0.004
APACHE II score	30 (23,37)	29 (22, 33)	35 (26, 39)	0.02

Data are presented as number (proportion) or median (interquartile range). Percentages may not sum to 100 due to rounding. *APACHE* Acute Physiology and Chronic Health Evaluation, *ARDS* acute respiratory distress syndrome, *CKD* chronic kidney disease, *COPD* chronic obstructive pulmonary disease. *Comparing survivors and non-survivors (Chi-squared likelihood test for categorical variables and Wilcoxon rank sum test for continuous variables). **Median (IQR) estimated glomerular filtration rate at admission = 47.6 (25.1, 88.3) mL/min/1.73 m^2^ in *n* = 60 patients in the cohort

### Metabolome in sepsis

**Fig. 1:**
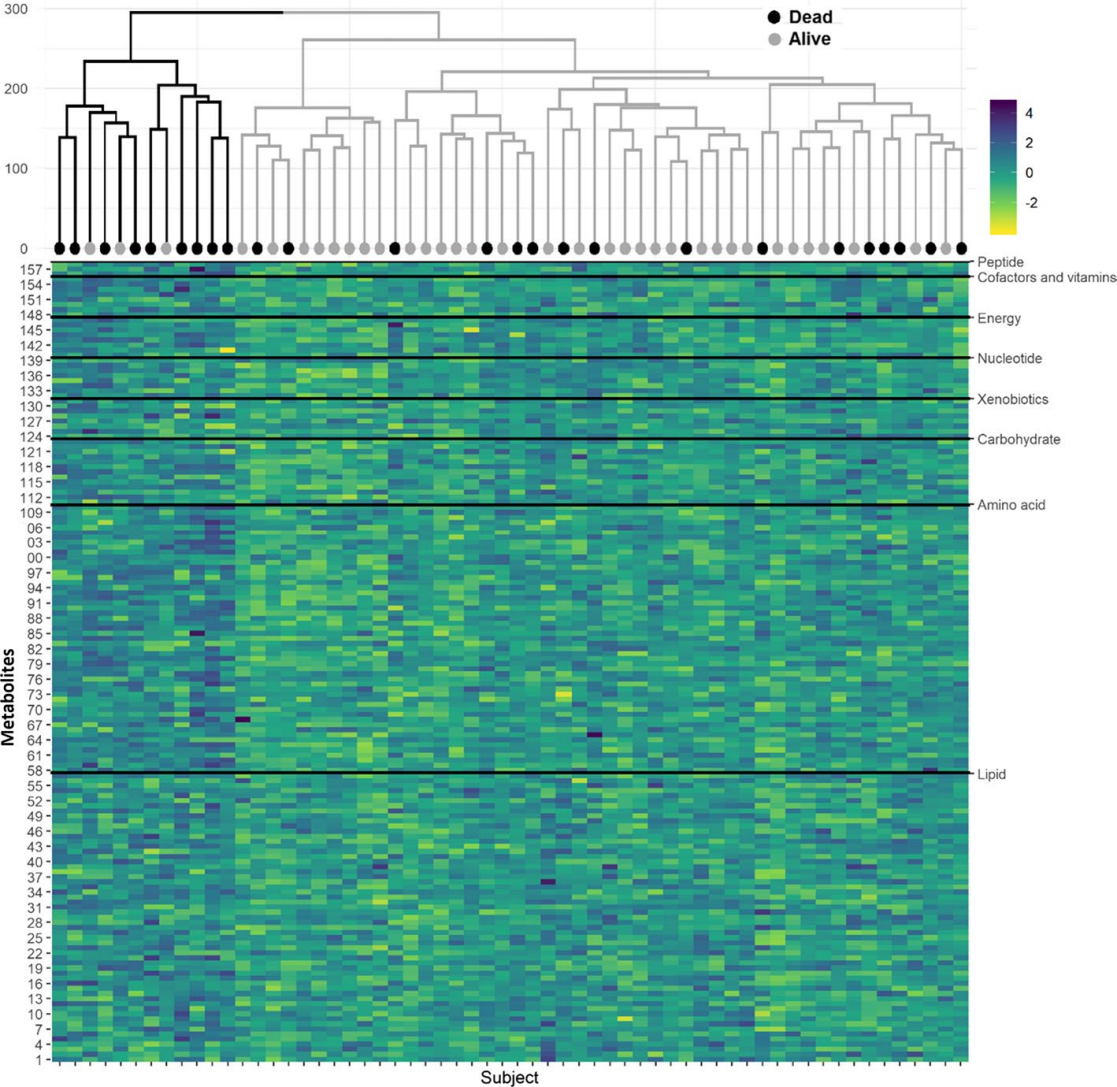
Heatmap showing normalized metabolite levels grouped by super-pathway among patients with sepsis. Heatmap showing normalized metabolite levels (rows), grouped by super-pathway, among patients with sepsis following hierarchical clustering (dendrogram, top); survival status is annotated (grey = yes, black = no). Differences in the metabolome among survivors and non-survivors were noted across multiple metabolic super-pathways

A total of 158 metabolites passed quality control and pre-processing filters and were included in the analysis, representing 8 super-pathways (Additional file 1: Table S1). Overall differences in the metabolome were observed among survivors and non-survivors (Fig. 1). Twenty principal components were required to explain 80% of the variance in the metabolome (Additional file 1: Figs. S1 and S2).

### Model training and performance

**Fig. 2:**
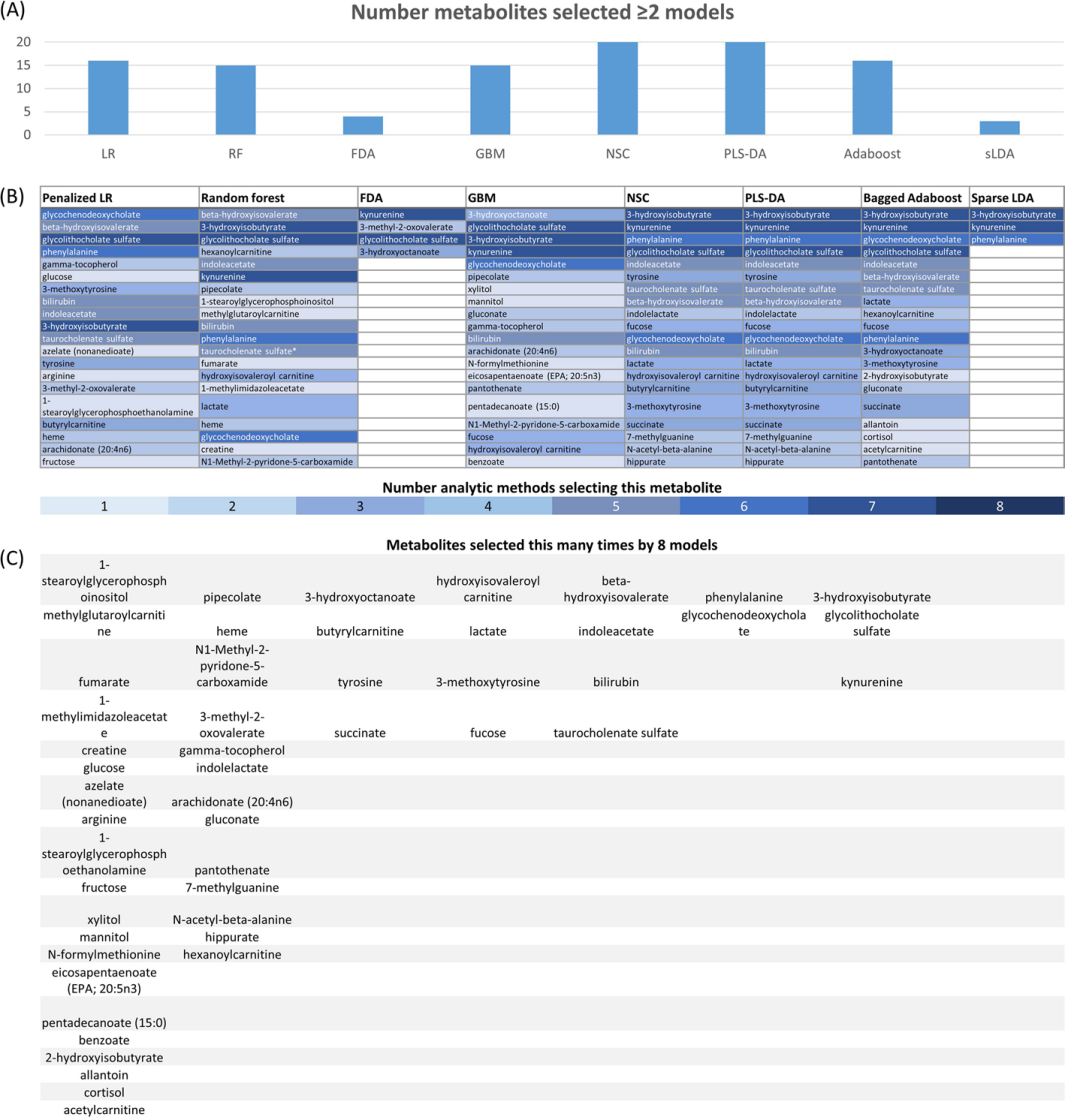
Comparison of top metabolites selected by each analysis method’s innate feature selection algorithm. Comparison of top metabolites selected by each analysis method’s innate feature selection algorithm, identifying metabolites that more meaningfully contribute to successful sepsis mortality prediction models. Such approaches may identify measures of association and individual metabolic links with mortality. Agreement was noted between the lists of top metabolites identified by several machine learning methods, which also overlapped with those identified by conventional panelized logistic regression. *FDA* flexible discriminant analysis, *GBM* generalized boosted regression models, *LR* logistic regression, *NSC* nearest shrunken centroids, *PLS-DA* partial least squares-discriminant analysis, *sparse LDA* sparse discriminant analysis

Model specificity (for 28-day mortality) ranged from 0.619 to 0.821 (Additional file 1: Table S2). Specificity of survival status (that is, the ability of a model to correctly identify people who did not die) was highest for PLS-DA. Several metabolites were consistently prioritized by multiple ML methods (Fig. 2a, b), which are also separately categorized by metabolic super-pathway (Additional file 1: Fig. S3). PLS-DA and nearest shrunken centroids prioritized the greatest number of metabolites identified by at least one other method. Logistic regression and random forests selected a similar proportion of prioritized metabolites. Sparse linear and flexible discriminant analyses identified the fewest metabolite contributors to mortality prediction, although the metabolites ranked were frequently ranked by multiple other methods. Logistic regression models penalized for multiple hypothesis testing demonstrated consistent results with many ML methods. The metabolites ranked the most frequently by multiple models included lactate, bile acid metabolites (glycolithocholate sulfate and glycochenodeoxycholate) and amino acid metabolism (kynurenine [tryptophan metabolism], 3-hydroxyisobutyrate [valine metabolism], and phenylalanine) (Fig. 2c). Receiver operating characteristic (ROC) curves for the assessment of each ML model’s performance are included in Additional file 1: Fig. S4.

### Metabolites linked with survival

Across the metabolome, the 13 metabolites with the strongest linkage to mortality defined through an ensemble importance score (representing the consistency and strength with which a metabolite was highly selected across all the ML models) ≥ 0.5 included lactate, bilirubin, kynurenine, glycolithocholate sulfate, glycochenodeoxycholate, indoleacetate, phenylalanine, 3-hydroxyisobutyrate, beta-hydroxyisovalerate, taurocholenate sulfate, 3-methoxytyrosine, fucose, and hydroxyisovaleroylcarnitine (Table 2, Additional file 1: Fig. S5). These top metabolites are linked with the tryptophan, pyruvate, phenylalanine, pentose phosphate, and bile acid metabolic pathways. Distributions of actual and imputed datasets are provided for each metabolite in Additional file 1: Table S3.

**Table 2:** Top-ranked metabolites linked with survival ranked by ensemble machine learning-derived summary importance score (defined as those with importance score ≥ 0.5), with corresponding median (interquartile range) normalized levels among septic patients who survived (*N* = 35) and those who died (*N* = 25)

Metabolite	Ensemble metabolite importance score	Median (IQR) normalized level among sepsis patients who survived (*N* = 35)	Median (IQR) normalized level among sepsis patients who died (*N* = 25)	*p* value
3-Hydroxyisobutyrate^1^	0.875	252,438.3 (205,768.5–330,512.0)	447,733.7 (317,285.8–649,937.2)	< 0.001
Glycolithocholate sulfate^1^	0.875	25,510.2 (10,242.8–134,807.9)	79,337.8 (43,853.3–180,873.5)	0.013
Kynurenine	0.875	1,210,710.5 (842,693.3–1,538,991.4)	2,157,986.3 (1,661,243.8–3,509,181.0)	< 0.001
Glycochenodeoxycholate	0.75	272,981.5 (167,008.1–465,149.2)	732,421.1 (438,467.2–1,813,588.0)	0.007
Phenylalanine	0.75	32,946,718 (28,021,921–39,246,282)	43,833,240 (35,133,532–66,127,232)	0.001
Beta-hydroxyisovalerate	0.625	144,346.7 (99,003.9–176,673.7)	211,728.6 (136,285.5–467,855.3)	0.006
Bilirubin	0.625	49,090.9 (26,390.4–76,850.4)	114,271.2 (48,093.5–529,768.6)	0.01
Indoleacetate^1^	0.625	74,409.6 (51,108.5–84,809.9)	102,489.8 (81,114.9–136,096.5)	0.002
Taurocholenate sulfate	0.625	102,518.9 (50,764.5–297,900.4)	468,138.2 (101,984.3–639,836.3)	0.009
3-Methoxytyrosine	0.5	65,256.5 (50,705.4–74,069.8)	72,094.6 (58,031.8–96,055.7)	0.024
Fucose^1^	0.5	148,841.4 (80,563.7–216,589.1)	260,624.5 (134,398.9–312,514.6)	0.004
Hydroxyisovaleroylcarnitine^1^	0.5	127,932.2 (81,047.1–183,819.4)	237,218.2 (107,023.4–276,240.9)	0.024
Lactate	0.5	91,420,816 (60,458,968–113,868,176)	147,947,760 (76,580,752–235,829,728)	0.012

^1^Of these top identified metabolites, 4/13 (3-hydroxyisobutyrate, glycolithocholate sulfate, indoleacetate, and fucose) have not been previously correlated with sepsis survivorship. The remaining 9 metabolites have been previously identified in metabolomic studies in this and other cohorts.^12,13^

## Discussion

The complementary objectives of this study were: (1) biologically, to uncover metabolites associated with sepsis mortality; and (2) methodologically, to evaluate ML methods as tools to support metabolite selection and prioritization in association studies, comparing their performance to that of conventional statistical methods. Across the broad metabolome profiled, top “hits” identified by ML methods included metabolites with well-established clinical importance (including lactate and bilirubin), as well as metabolites with less established links to sepsis outcomes (including those relating to tryptophan, pyruvate, phenylalanine, pentose phosphate, and bile acid metabolism). An integrated, ML-based ensemble ranking method for prioritizing metabolites based on the strength and consistency of their linkage with survival provided robust metabolite rankings. The ensemble method combined the ranking results of multiple training models in order to obtain a robust set of important metabolites. Overall, these findings support that the application of ML methods metabolomics data may support and possibly enhance biologic discovery, even in small cohorts of critically ill patients; further studies in larger cohorts will be needed to generalize these results**.**

Sepsis is a major catabolic insult that results in profound changes in carbohydrate, fat, and amino acid metabolism [17, 18]. Several metabolites are routinely used in clinical practice for prognostication and treatment decisions, including lactate [19, 20]. Serum lactate may rise in sepsis due to mitochondrial dysfunction, adrenergic signaling, impaired hepatic metabolism, and tissue hypoxia and resultant anaerobic respiration [21–23]. The prioritized identification of lactate as a “top” metabolite by the ensemble consensus ranking procedure herein provides an important marker of external validity (effectively, a positive control for the statistical methods). Similarly, levels of bilirubin and bile salt metabolites are increased in sepsis and have been associated with increased mortality; proposed mechanisms include attenuated bile-acid transporter production and hepatic hypoperfusion, as well as enhanced cytokine and nitric oxide production with subsequent inflammation-mediated cholestasis [24, 25]. Our findings consistently demonstrated strong links between numerous other biliary pathway metabolites and sepsis survivorship. In addition, our study corroborated previously documented associations of several amino acid pathway metabolites with sepsis survivorship, including kynurenine (a downstream tryptophan metabolite) and phenylalanine [12, 13]. Elevated kynurenine levels have been previously identified as a predictor of the development of sepsis in trauma patients, and have also been associated with impaired vascular microreactivity, hypotension, and immune dysregulation in sepsis [26–29]. Alterations in tryptophan metabolism have also been documented in other inflammatory conditions, such as cardiac arrest, ischemia–reperfusion injury, systemic lupus erythematosus, and COVID-19 [30–33], suggesting that this association may be representative of an broad role for tryptophan metabolism in diseases of dysregulated inflammation. Elevated phenylalanine levels have similarly been shown to both predict the development of sepsis and correlate with increased mortality [12, 13, 23, 34]. Our study provides important clinical corroboration of these findings through data-driven molecular and statistical methods. Future studies are needed to determine to what extent these metabolites may be markers versus causal mediators of outcomes in sepsis.

Moreover, our study identified four metabolites (3-hydroxyisobutyrate, glycolithocholate sulfate, indoleacetate, and fucose) not been previously linked with sepsis survival. 3-hydroxyisobutyrate is a catabolic byproduct of valine metabolism, and elevated levels have been previously linked with insulin resistance and diabetes [35, 36]. Stress hyperglycemia and glycemic variability are associated with sepsis mortality, and accordingly this link may provide further corroboration and insight into this interaction [37, 38]. Fucose is known to play a role in the potentiation of leukocyte adhesion and lymphocyte homing, as well as an inhibitory role in antibody-mediated cellular cytotoxicity [39]. This interaction with both innate and adaptive immunity may play a role in the association of higher fucose levels with poor sepsis survivorship. Lastly, elevations in glycolithocholate sulfate, a secondary bile acid metabolite, and indoleacetate, a tryptophan byproduct, among non-survivors may relate to the complex patterns of bilirubin and tryptophan metabolism in sepsis as described above. ML methods may hence present an important tool for the discovery of new targets to improve sepsis care and better understand pathophysiology.

This current report builds upon a prior analysis from this cohort which had used conventional logistic regression, as well as a network-based approach [12]. One of the key objectives in the current study was to examine whether ML methods could improve the detection of metabolite associations beyond conventional statistical methods. Indeed, 4 of the top 13 metabolites identified by ML prioritization herein had not previously been linked with sepsis survival, while the remainder corroborated this previously work. Conventional statistical approaches may have limitations when applied to large, complex biologic systems such as the metabolome. Many of these limitations are due to the high degree of internal correlation structure, subclass heterogeneity, and missingness inherent in the study of interconnecting biologic pathways and processes [40]. The theoretical advantages of ML strategies include the ability to flexibly integrate multiple forms of data analysis, enhance complex pattern recognition, classify high-order metabolite–metabolite interactions and manage internal correlation, and better address high dimensionality and small sample size of data [41, 42], Each of these advantages may enable ML methods to support deeper interrogation of the metabolome as a complete system. Indeed, ML methods have been used broadly in clinical risk prediction, including in sepsis [12, 13] and other diseases such as cardiovascular disease [11]. However, beyond this role in building accurate risk prediction models, ML methods may also support biologic target discovery and prioritization—as we evaluated herein. This latter application has been demonstrated in functional and regulatory genomics, tumor biomarker discovery, and evolutionary population genetics [43–45]. Overall, our results support the use of feature selection tools innate to AI/ML methods for biologic discovery, demonstrating internally consistent and biologically plausible results. Moreover, the ensemble method used herein may be useful as a composite ranking procedure for target prioritization.

These findings should be considered hypothesis-generating in view of several potential limitations. First, patients with sepsis were selected for this exploratory study on the basis of interleukin-18 levels as well as based on having concurrent ARDS or not, which may limit external generalizability, as may the incidentally high proportion of participants with comorbid malignancy in this cohort. Other relevant clinical variables which were not available for assessment include nutritional status and ARDS severity. Given that these clinical features may influence the metabolome of sepsis patients as well as confound the observed metabolic patterns specific to survivors, these results require validation in external cohorts. Additionally, the observed associations of metabolites with survival may not be a specific feature of sepsis; without a control group, these associations may be a broader feature of critical illness or infection. Further studies with appropriate controls will be necessary to delineate this. Similarly, the final list of metabolites linked with survival depends on the selection of metabolites included in the platform; however, the 158 metabolites that passed quality control and pre-processing filters and were included in the primary analysis represented a diversity of biology functions and pathways potentially relevant in sepsis. Furthermore, sepsis [46–50], ARDS [51], and other critical illnesses [52] are increasingly recognized as heterogeneous conditions with potentially important subclasses. Our cohort was too small to stratify by the selection variables or by potential subclasses. Second, numerous, non-physiologic variables contribute to clinical course in the ICU, including patients’ and surrogate decision-makers’ preferences around the provision of life-sustaining care [53, 54], potentially limiting biologic association studies. Third, given that the purpose of our analysis was to identify potential metabolic mediators of sepsis outcomes, and not to build clinical prediction tools [12], we intentionally did not adjust for clinical variables, including illness severity. Worse illness severity may mediate the associations between metabolites and mortality, and adjusting for collinear variables would potentially attenuate associations and limit discovery. Future studies may build upon our exploratory results to establish these metabolic associations in larger cohorts and utilize ML for the purpose of clinical risk prediction. Fourth, plasma was collected within 72 h of ICU admission; this window may be long in view of the rapidity of the disease course in sepsis. Finally, as noted above, we did not undertake external validation of the findings in an independent cohort. While a number of the metabolites linked with sepsis mortality herein are previously known, lending some support to external validity, further investigation is required particularly in the validation of the four new metabolite associations identified. Given the limitations discussed above in the size, selection, and clinical features in this exploratory analysis, further validation in an external cohort is required in order to increase the generalizability and ascertainment of validity of these results.

## Conclusions

In this modest-sized cohort of critically ill patients with sepsis, application of multiple artificial/machine learning methods supported identification of metabolites associated with clinical outcomes. While these hypothesis-generating results require validation in external cohorts, such metabolites and metabolic pathways may represent new diagnostic, prognostic, or therapeutic targets. Advancing an understanding of these approaches will be critical in fostering such robust methods of biologic discovery in critical illness.

## Supplementary Information

**Additional file 1: Table S1.** Super-pathways represented by the 158 metabolites passing quality control and pre-processing filters. **Table S2.** Performance measures of machine learning algorithms trained under the precision recall (PR) curve in discriminating survival status using metabolomics data. **Table S3**. Variable distributions in complete imputed dataset and without imputation. **Figure S1. A** Total variance explained by each principal component and [middle and bottom panels] cumulative variance explained by each component (in pink) shown with the cross-validated variance explained (in blue). **B** Twenty principal components are required to explain 80% of the variance in the data. **Figure S2. A** Plot of the first 2 principal components. Ellipse captures 95% of the data. **B** Metabolites contributing to the largest loading weights for the first and second PC. **Figure S3.** Super pathways represented among top metabolites ranked by machine learning approaches. **Figure S4**. ROC curves for models in Table S2.** Figure S5.** Pairwise comparisons of normalized top metabolite levels, stratified by survival status. (Figure separated for data visualization purposes only.)

## Data Availability

Data can be provided by the corresponding author upon request.
