# Supplementary material for: Machine learning approaches to the human metabolome in sepsis identify metabolic links with survival
Source: Intensive Care Med Exp. 2022 Jun 17;10:24. doi: 10.1186/s40635-022-00445-8 (PMC9203139; doi:10.1186/s40635-022-00445-8)
Supplement: Supplementary file 1 — Additional file 1: Table S1. Super-pathways represented by the 158 metabolites passing quality control and pre-processing filters. Table S2. Performance measures of machine learning algorithms trained under the precision recall (PR) curve in discriminating survival status using metabolomics data. Table S3. Variable distributions in complete imputed dataset and without imputation. Figure S1. A Total variance explained by each principal component and [middle and bottom panels] cumulative variance explained by each component (in pink) shown with the cross-validated variance explained (in blue). B Twenty principal components are required to explain 80% of the variance in the data. Figure S2. A Plot of the first 2 principal components. Ellipse captures 95% of the data. B Metabolites contributing to the largest loading weights for the first and second PC. Figure S3. Super pathways represented among top metabolites ranked by machine learning approaches. Figure S4. ROC curves for models in Table S2. Figure S5. Pairwise comparisons of normalized top metabolite levels, stratified by survival status. (Figure separated for data visualization purposes only.) [file 40635_2022_445_MOESM1_ESM.docx]

**Additional file 1 METHODS, TABLES, AND FIGURES**

**Machine Learning Approaches to the Human Metabolome in Sepsis Identify Metabolic Links with Survival**

Kosyakovsky, Somerset *et al.*

**Additional file 1 Methods**

**Data Pre-Processing:** Several steps were taken to pre-process the metabolomics data. The original data set contained 411 predictors. There were 244 (59.4%) metabolites with missing observations. At a 5% Bonferroni-corrected significance level, there was no difference in the proportion of missing values between the two survival groups in any metabolites. We removed 155 metabolites with >10% missing data. Prior to imputation, metabolite observations were centered to have median equal to 1. Assuming that missing observations may reflect metabolites with concentrations below the chemical detection limit, missing values in each metabolite were imputed to the new minimum. Metabolite observations were log10 transformed to approximate a normal distribution and scaled if required by ML algorithms. To support dimension reduction, we employed an algorithm that identified the minimal set of metabolite predictors such that all the absolute pairwise correlations were below 0.75. If two variables had a high correlation, we determined which one was involved with the most pairwise correlations and this metabolite was removed. This removed 98 metabolites. Scores and loading plots from principal components analysis were examined to visualize the remaining data structure (**Supplemental Figures 1 and 2**).

**Feature Selection:** We used ML methods predicting mortality to identify the individual metabolites with strongest relationship to death. We applied ML methods (random forests, support vector machines, random *k*-nearest neighbors, nearest shrunken centroids, adaptive bagging/boosting, LASSO regression), conventional bilinear factor models (partial least squares regression-discriminant analysis), as well as traditional penalized (Benjamini-Hochberg and FDR) logistic regression models, predicting mortality using the post-processed metabolomics data. Models were trained under precision recall (PR) curves – which may be a better diagnostic tool for classification models trained on imbalanced datasets (J. Davis and M. Goadrich, "The relationship between Precision-Recall and ROC curves.," Proceedings of the 23rd International Conference on Machine learning., no. ACM, pp. 233-240, 2006 – using 50 repeats of 5-fold cross validation. Innate feature selection and ranking tools of ML were used to prioritize metabolites. The top 20 metabolites contributing to successful predictive model generation were compared across each method. An ensemble method for hybridizing ML methods to run in series was applied, and the variables with ensemble importance scores ≥0.5 were examined. All analysis was conducted in R *v*3.3.2 using packages caret (6.0-79), earth (4.6.2) , spls (2.2-2), klaR, randomForest (4.6-14), RWeka (0.4-38), fastAdaboost (1.0.0), adabag (4.2), plyr (1.8.4), sparseLDA (0.1-9), glmnet (2.0-16), Matrix (1.2-14), gbm (2.1.3), and pamr (1.55). Additional notes about ML methods included:

- *Random Forests (RF)*

This model has implicit feature selection (therefore resistant to non-informative or redundant predictors) and can produce interpretable results. Additionally, the model is resistive to outliers and can specifically account for missing data. Variable importance was determined according to the mean decrease in accuracy.^1^

The number of randomly selected variables at each split was varied over 1 to 24 and the number of trees was set to 500.

- *Boosted Trees (BT)*

The depth of the tree ranged sequentially from 1 to 3 and the number of boosting iterations ranged from 50 to 150 in 50 iteration increments. Variable importance was defined using the relative influence.^2^

- *Penalized logistic regression (PLR)*

The model has implicit feature selection (therefore resistant to non-informative or redundant predictors) and produces interpretable results. The downside to the scaling and centering transformation is a loss of interpretability since the data is no longer in original units. This model is known to have poor results for high-dimensional data (n<p).

The candidate values for the mixing percentage is 0 to 1 by increments of 0.2 and the regularization parameter is varied from 0.01 to 0.2 by 0.05 increments. Since PLR imposes a penalty term for too many variables in the model, it will shrink less important variables towards zero. Therefore, predictors are ranked according to the size of their estimated regression coefficients.

- *Flexible discriminant analysis (FDA)*

Allows n<p, can create non-linear classification boundaries and depending on the tuning parameters, it can be easily interpreted. It performs automatic feature selection and is not adversely affected when a large number of non-informative predictors are used as inputs. Moreover, correlated predictors do not drastically affect model performance, but they can complicate model intepretation.

We performed a flexible discriminant analysis and used multivariate adaptive regression splines (MARS) in the regression stage. This is a suitable approach for problems with high input dimensions. It does not assume or impose any particular type of relationship between the predictor variables and the dependent outcome. The MARS algorithm has 2 parameters, the degree (1 – additive model, 2- pairwise interactive model) and the pruning number. MARS uses the pruning technique to counteract overfitting and hence, improve the predictive capability of our final model. In the selection of and pruning of basis functions, the MARS algorithm is naturally selecting a subset of predictors– making it a powerful tool.

Candidate values of the tuning parameters, degree and pruning numbers, ranged from 1-2.

- *Flexible descriminant analysis using multivariate adaptive regression splines, sparse linear discriminant analysis and partial least squares discriminant analysis*

The number of variables varied sequentially from 1 to 24 and the regularization parameter varied from 0.01 to 0.2 by increments of 0.0475.

- *Partial least squares discriminant analysis (PLS-DA)*

If predictors are highly correlated or exceed the number of samples collected, usual discriminant analysis cannot be used. This model is often used to identify metabolites that best discriminate samples from different groups. This is a suitable choice if complex correlation structure exists and as a result, one wants to avoid unsupervised correlation filter preprocessing steps. Moreover, it has a model-specific variable importance metric based on the weighted sums of the regression coefficients.

- *Nearest shrunken centroids*

Candidate values for the shrinkage threshold parameter are 300 to 3. A metabolite is condidered useful to classification if its observations have low variance within the same class but are largely different to its values in a different class.^3^

**Additional file 1 Tables and Figures**

**Additional file 1 Table 1:** Super-pathways represented by the 158 metabolites passing quality control and pre-processing filters.

| Super pathway | # of predictors |
| --- | --- |
| Amino Acid | 53 |
| Carbohydrate | 13 |
| Cofactors and vitamins | 8 |
| Energy | 8 |
| Lipid | 57 |
| Nucleotide | 8 |
| Peptide | 3 |
| Xenobiotics | 8 |

**Additional file 1 Table 2:** Performance measures of machine learning algorithms trained under the precision recall (PR) curve in discriminating survival status using metabolomics data.

| Model | AUPRC* | AUROC* | Sensitivity* | Specificity* | Brier Score* |
| --- | --- | --- | --- | --- | --- |
| NSC | 0.793 | 0.800 | 0.689 | 0.818 | 0.190 |
| PLS-DA | 0.786 | 0.798 | 0.678 | 0.821 | 0.196 |
| Random Forest | 0.769 | 0.774 | 0.681 | 0.741 | 0.200 |
| Bagged AdaBoost | 0.729 | 0.744 | 0.687 | 0.659 | 0.207 |
| GBM | 0.725 | 0.735 | 0.645 | 0.723 | 0.209 |
| Penalized LR | 0.688 | 0.705 | 0.622 | 0.674 | 0.215 |
| FDA | 0.660 | 0.684 | 0.582 | 0.675 | 0.291 |
| Sparse LDA | 0.642 | 0.655 | 0.630 | 0.650 | 0.248 |

**Abbreviations:** AUPRC = area under the precision recall curve; AUROC = area under the receiver operator characteristic score; FDA = flexible discriminant analysis; GBM = generalized boosted regression models; LR = logistic regression; NSC = nearest shrunken centroids; partial least squares-discriminant analysis. *AUPRC and AUROC correspond to the area under the average ROC curve and average PR curve over cross-validation folds. *****Sensitivity and specificity correspond to the point on the average ROC curve closest to optimal sensitivity and specificity (1,1). *The Brier score is a score function that measures the accuracy of probabilistic predictions, with lower scores indicating better calibration.

**Additional file 1 Table 3**: Variable distributions in complete imputed dataset and without imputation.

| **Metabolite** | **Proportion (%) imputed** | **Dataset with Imputation** | | **Dataset without Imputation** | |
| --- | --- | --- | --- | --- | --- |
|  |  | **Median** | **IQR** | **Median** | **IQR** |
| 3-hydroxyisobutyrate | 0.00% |  |  |  |  |
| glycolithocholate sulfate | 8.33% | 0.72 | 0.26-2.27 | 1.00 | 0.31-2.56 |
| kynurenine | 0.00% |  |  |  |  |
| glycochenodeoxycholate | 3.33% | 0.98 | 0.42-2.33 | 1.00 | 0.45-2.55 |
| phenylalanine | 0.00% |  |  |  |  |
| beta-hydroxyisovalerate | 0.00% |  |  |  |  |
| bilirubin | 1.67% | 0.97 | 0.51-2.12 | 1.00 | 0.52-2.15 |
| Indoleacetate | 3.33% | 1.00 | 0.81-1.43 | 1.00 | 0.83-1.45 |
| taurocholenate sulfate | 5.00% | 0.91 | 0.37-3.26 | 1.00 | 0.49-3.30 |
| 3-methoxytyrosine | 1.67% | 0.99 | 0.77-1.2 | 1.00 | 0.78-1.21 |
| fucose | 3.33% | 0.97 | 0.52-1.58 | 1.00 | 0.59-1.59 |
| Hydroxyisovaleroyl carnitine | 10.00% | 0.83 | 0.48-1.48 | 1.00 | 0.56-1.56 |
| lactate | 0.00% |  |  |  |  |

**Additional file 1 Figure 1:** **[A]** Total variance explained by each principal component and [middle and bottom panels] cumulative variance explained by each component (in pink) shown with the cross-validated variance explained (in blue). **[B]** Twenty principal components are required to explain 80% of the variance in the data.

**[A]**


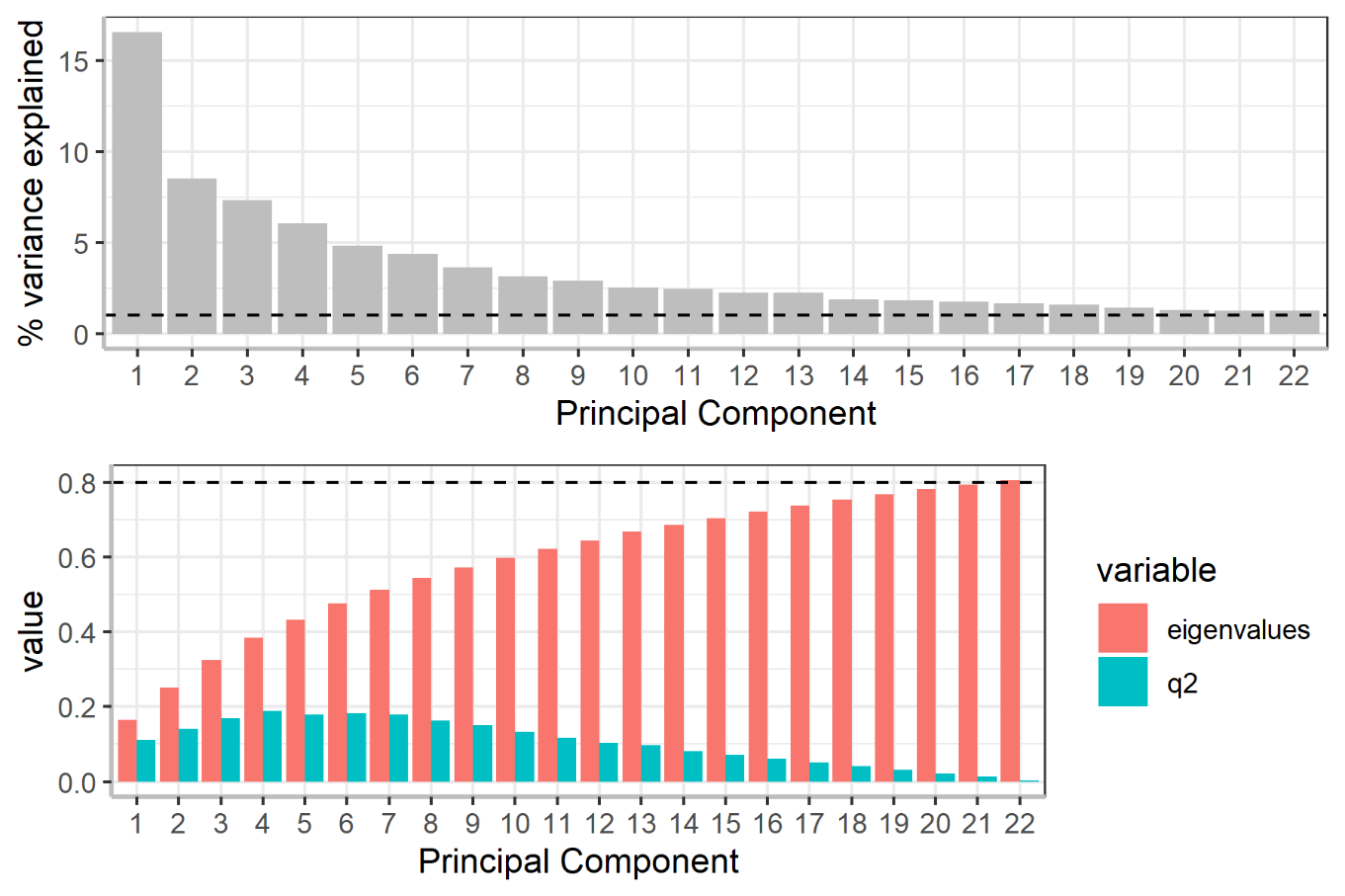


**Figure 1 (cont.)**

**[B]**

**
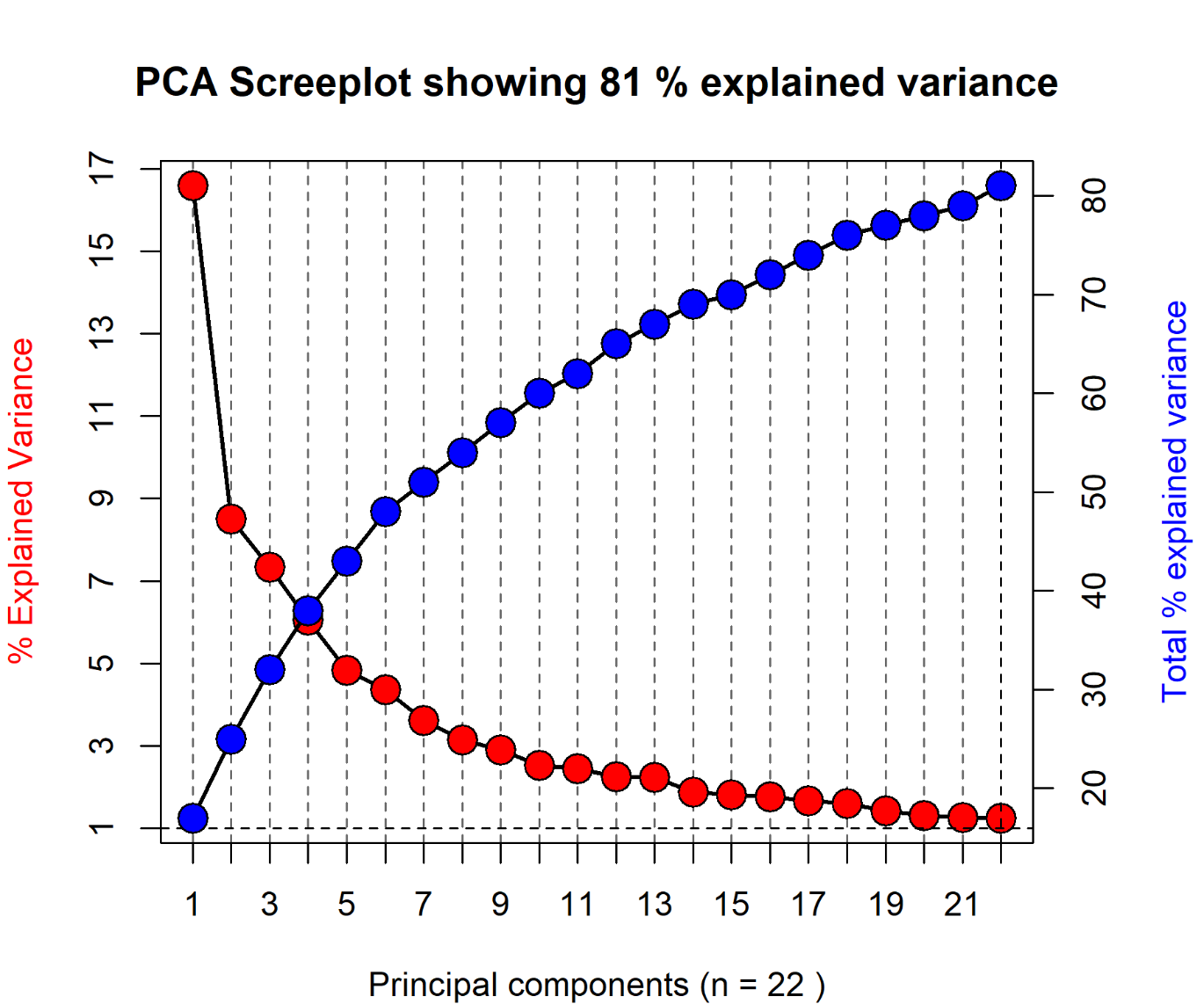
**

**Additional file 1 Figure 2:** **[A]** Plot of the first 2 principal components. Ellipse captures 95% of the data. **[B]** Metabolites contributing to the largest loading weights for the first and second PC.

**[a]**


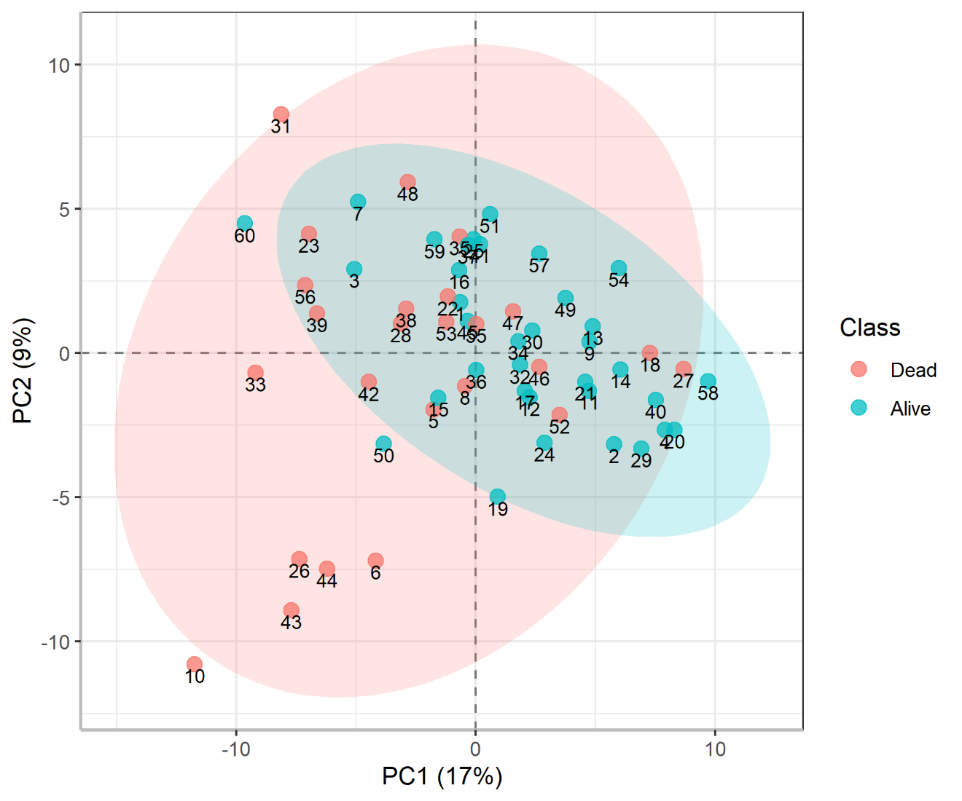


**[b]**


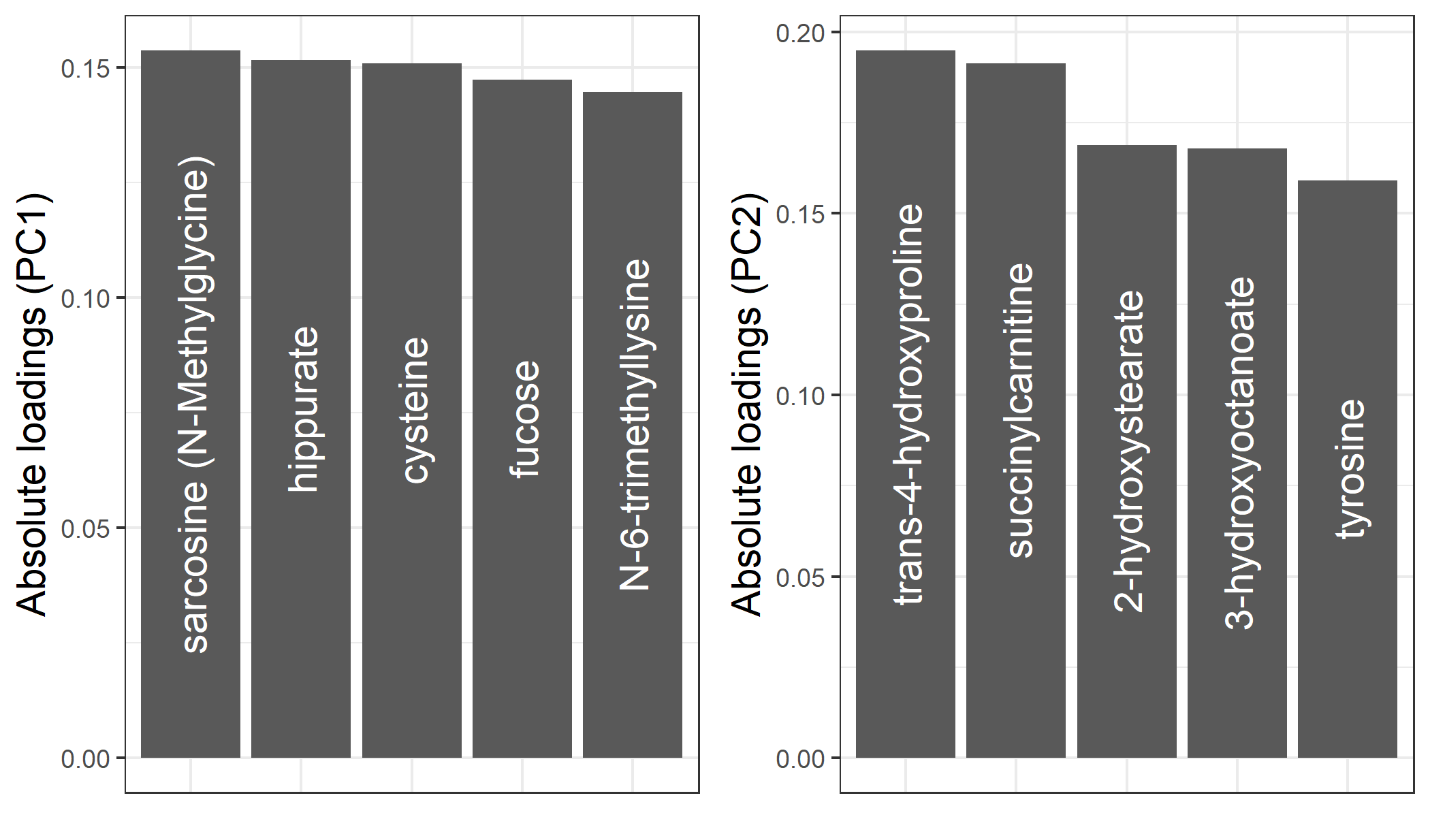


**Additional file 1 Figure 3:** Super pathways represented among top metabolites ranked by machine learning approaches.


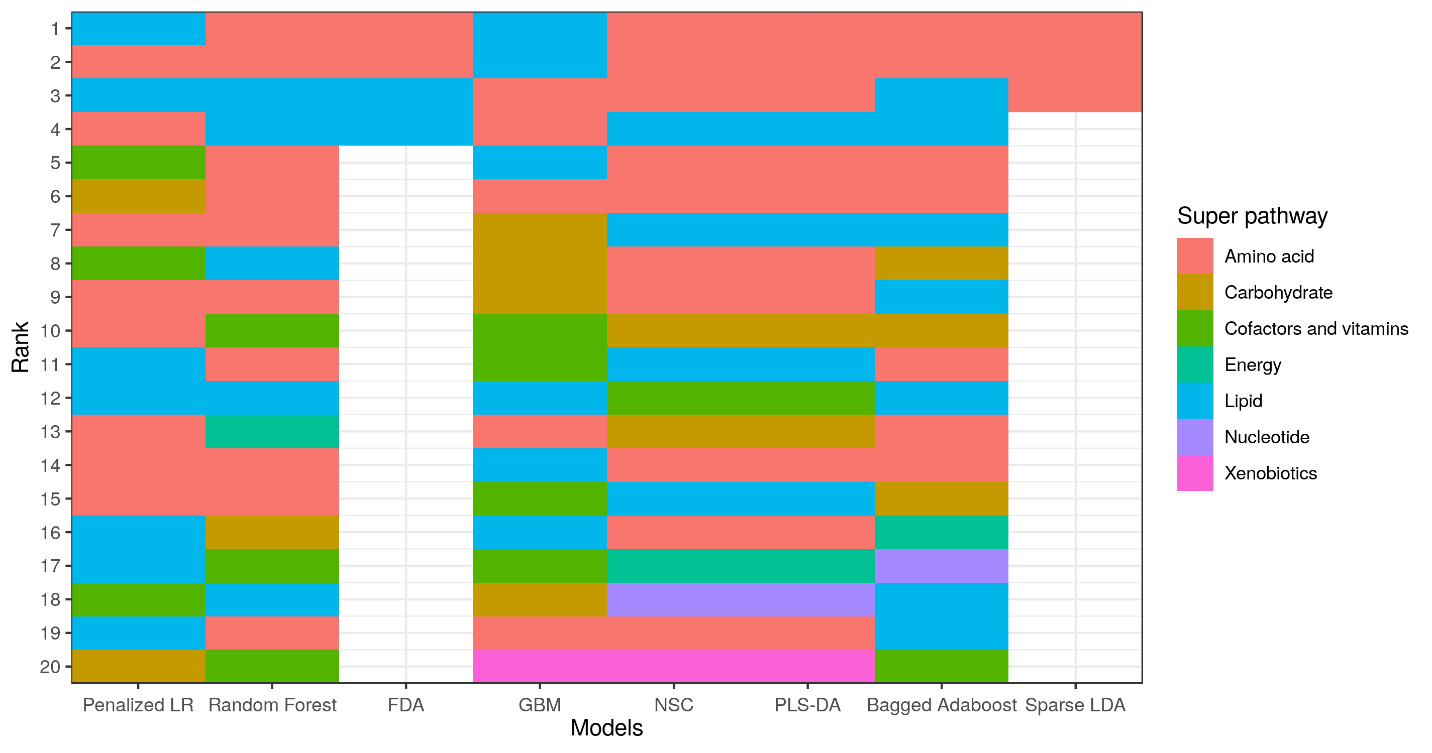


**Additional file 1 Figure 4**: ROC curves for models in **Additional file 1 Table 2**.


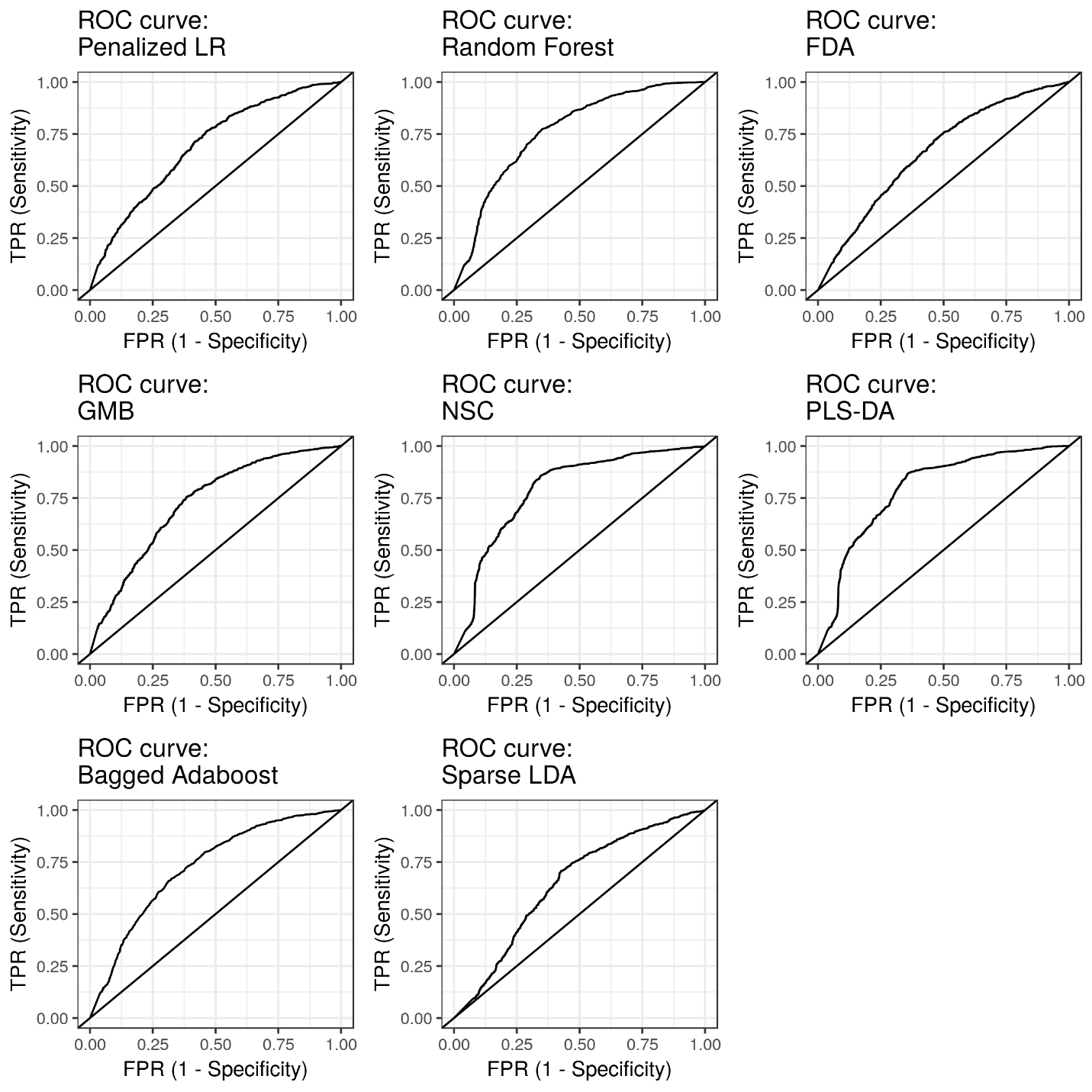


**Additional file 1 Figure 5:** Pairwise comparisons of normalized top metabolite levels, stratified by survival status. (Figure separated for data visualization purposes only.)

**
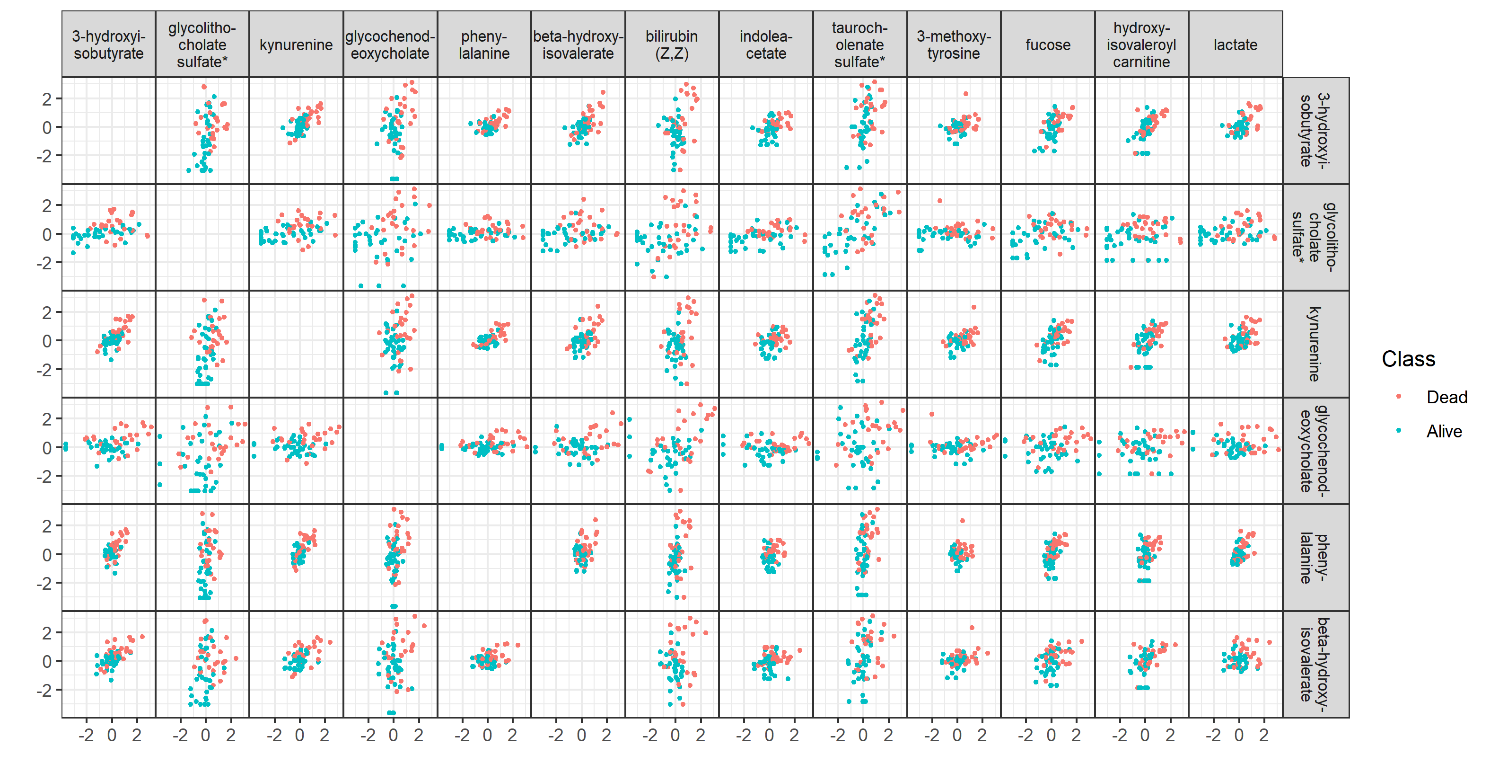

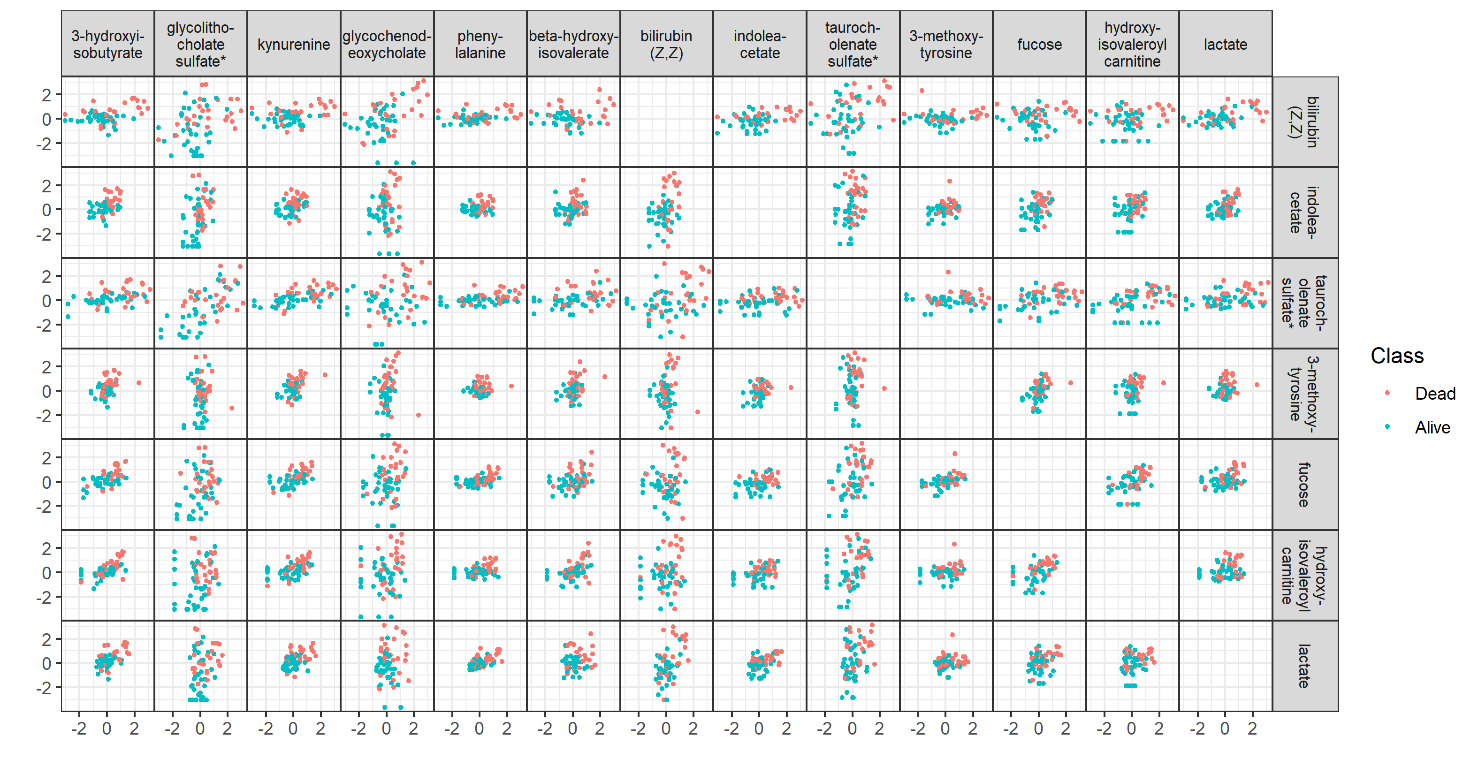
**

**Additional file 1 References:**

1. Breiman, Leo (2001) "Random forests." Machine learning 45.1: 5-32.
2. Friedman, Jerome H (2001) "Greedy function approximation: a gradient boosting machine." Annals of statistics 1189-1232.
3. Tibshirani, Robert, et al (2002) "Diagnosis of multiple cancer types by shrunken centroids of gene expression." Proceedings of the National Academy of Sciences 99.10: 6567-6572
